# BMAL1 may be involved in angiogenesis and peritumoral cerebral edema of human glioma by regulating VEGF and ANG2

**DOI:** 10.18632/aging.203708

**Published:** 2021-11-23

**Authors:** Fan Wang, CaiYan Li, Fei Han, LvAn Chen, Ling Zhu

**Affiliations:** 1 Department of Neurosurgery, The Affiliated Jingmen First People’s Hospital of Hubei Minzu University, Jingmen, China; 2 Department of Neurosurgery, The Second People’s Hospital of Jingmen, Jingmen, China; 3 Department of Oncology, The First Affiliated Hospital of Chongqing Medical University, Chongqing, China

**Keywords:** glioma, BMAL1, angiogenesis, HIF-1a, ANG2, VEGF

## Abstract

The rhythm gene *BMAL1* (Brain and Muscle ARNT-Like 1) may play an important role in glioma tolerance for anti-angiogenesis therapy. In humans with glioma of different pathological grades, *BMAL1* expression was significantly different, and the expression of *ANG2* (Angiopoietin 2) and *VEGF* (Vascular endothelial growth factor) was positively correlated with the expression of *BMAL1*. Additionally, *BMAL1* expression is positively correlated with the microvascular density and peritumoral edema of glioma. According to *in vitro* experiments, silencing the expression of *BMAL1* in primary glioma cells results in a decrease in the expression of *VEGF*. In contrast, overexpression of *BMAL1* promotes the expression of *ANG2* and *VEGF* via *HIF-1a* pathway. Therefore, *BMAL1* likely participates in the angiogenesis of glioma by modulating *ANG2* and *VEGF* expression, alters the therapeutic effect of anti-angiogenic treatments, and promotes peritumoral brain edema of glioma.

## INTRODUCTION

Glioma is the most common malignancy of the central nervous system, and it is highly invasive, results in a poor prognosis, and is prone to recurrence after surgery. Although conventional treatment for glioblastoma involves extensive radical resection combined with adjuvant radiotherapy and chemotherapy, the one-year cumulative survival rate of patients is still less than 30%. Only 3% of patients live longer than 5 years, with a total mean survival time of 6–9 months [1]. Therefore, studies aiming to determine the pathogenesis of glioma and identify methods to improve the clinical symptoms and prognosis of patients with glioma have become a hot topic in glioma research.

In recent years, vascular therapy targeting active angiogenic factors has become the standard treatment for some tumors, including colon cancer and kidney cancer [2, 3], and an adjuvant therapy for glioma [4, 5]. As glioma is an angiogenesis-dependent tumor with many heterogeneous vessels in the tumor tissue [6], vascular targeted therapy has great potential in the treatment of glioma. However, for the treatment of intracranial tumors, single anti-vascular therapy is far from satisfactory in achieving a therapeutic effect similar to that observed in other tumors [7]. In addition to the limitations of the drug itself, the most important limitation is the drug resistance of tumor cells [8, 9]. This tolerance may be related to rhythm genes [10].

In recent years, rhythm genes have been suggested to be related to the occurrence and development of various tumors, including breast cancer, colon cancer, and glioma [11–13]. As the core gene of rhythm gene, *BMAL1* is an important regulator that maintains normal cell and tissue homeostasis and plays an extremely critical role in tumor-related processes, such as cell proliferation and apoptosis, DNA repair, metabolism, and angiogenesis [14–17]. *BMAL1* is also considered a pro-tumor factor in glioma that promotes the proliferation and migration of glioma cells [18]. Therefore, an examination of the regulatory effects of BMAL1 on the expression of the angiogenic factors *ANG1* (Angiopoietin 1), *ANG2*, and *VEGF* in glioma will be conducive to understanding the reasons for glioma tolerance to vascular targeted therapy and the development of new target drugs. *HIF-1a* is an important regulatory factor of tumor angiogenesis. It was previously reported that *HIF-1a* can induce increased expression levels of *Ang2* and *VEGF* in hypoxia environment [19]. Therefore, in this study, we examined the correlation between the expression of the *BMAL1* gene and the expression of *HIF-1a*, *ANG1, ANG2*, and *VEGF* in human glioma tissues, as well as the relationships between the expression of the BMAL1 gene and the number of tumor microvessels and peritumor edema.

## RESULTS

### Differential expression of *BMAL1* and *HIF-1a* in human glioma tissue

**Figure 1:**
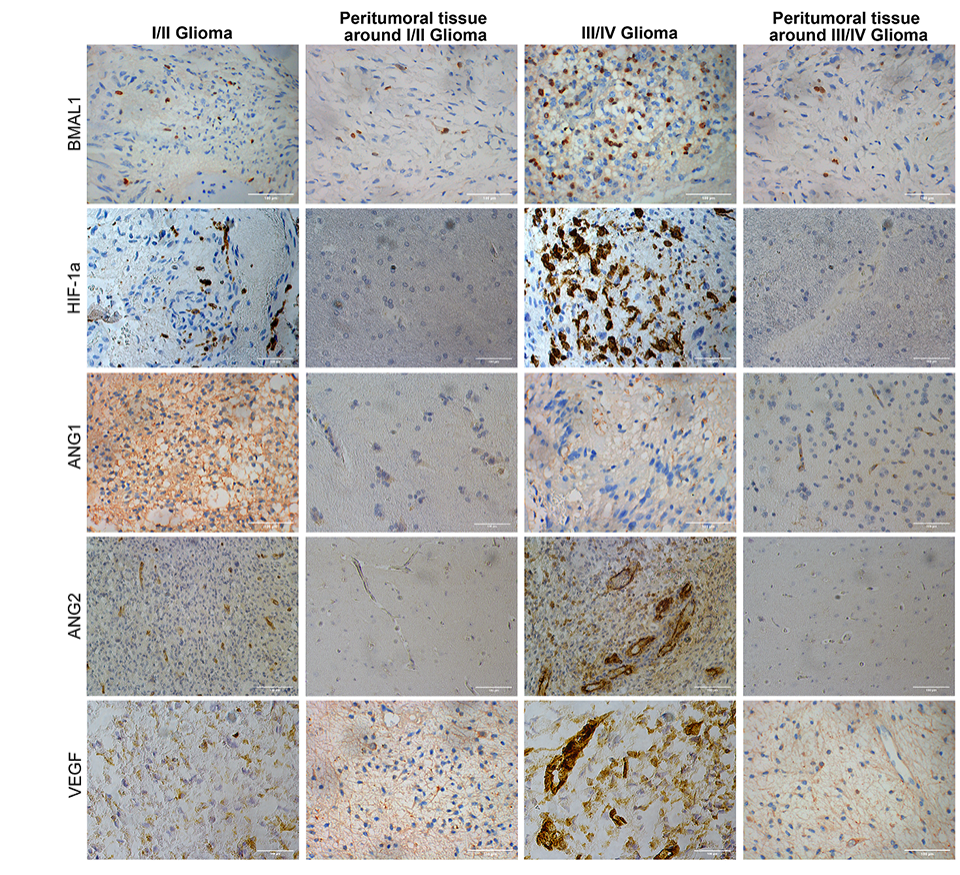
Immunohistochemistry analysis of BMAL1, HIF-1a, ANG1, ANG2, and VEGF expression in glioma and normal tissues with different pathological grades.

BMAL1 was expressed at different levels in both glioma and normal tissues (non-tumor brain tissue specimens), and all the staining was observed in the nucleus (Figure 1). The expression of BMAL1 was detected in 74.68% (59/79) of glioma tissues, and 31 (39.24%) of the 79 non-tumor brain tissues were positive for BMAL1. The expression level of BMAL1 in glioma cells was significantly different from non-tumor brain cells (*P* < 0.05) (Table 1).

**Table 1:** Comparison of BMAL1 positive expression between glioma and peritumoral tissues, and between different pathological grades and peritumoral tissues.

**Group**	***n***	**BMAL1 Positive**	***P***
Tumoral tissues Peritumor tissue	79 79	59 (74.68%) 39 (39.24%)	*P* < 0.05
I/II grades Peritumoral tissues around I/II grades	38 38	20 (52.63%) 17 (44.74%)	*P* = 0.491 > 0.05
III/IV grades Peritumoral tissues around III/IV grades	41 41	39 (95.12%) 22 (53.66%)	*P* < 0.001
I/II grades III/IV grades	38 41	20 (52.63%) 39 (95.12%)	*P* < 0.001

BMAL1 expression was not different between WHO grade low-grade gliomas (I/II) and adjacent non-tumor tissues (*P* > 0.05). The BMAL1 was expressed at significantly higher levels in high-grade glioma (III/IV) than in low-grade glioma (*P* < 0.001) and adjacent non-tumor tissues (*P* < 0.001) (Table 1). In addition, BMAL1 expression did not display significant relationships with age, gender, or tumor size (Table 2).

**Table 2:** Relationship between BAML1 expression and clinical data related to glioma.

**Sample**	***n***	**BMAL1 Positive**	***P***
**Age**			
<40	34	25 (73.53%)	
>40	45	34 (75.56%)	*P* = 0.819 > 0.05
**Sexual**			
Male	42	31 (73.81%)	
Female	37	28 (75.68%)	*P* = 0.827 > 0.05
**Tumor volume**			
<4 cm	45	34 (75.56%)	
>4 cm	34	25 (73.53%)	*P* = 1.000 > 0.05

Differential expression of HIF-1a was also observed between tumor tissues and peri-tumor tissues, and the positive expression was mainly concentrated in the nucleus (Figure 1). The expression of HIF-1a in high-grade gliomas was significantly higher than that in adjacent peritumor tissues (*P* < 0.001) and low-grade glioma (*P* < 0.01). Different from BMAL1 expression, HIF-1a expression in low-grade glioma tissues differs from that in adjacent peritumor tissues (*P* < 0.05).

### Expression of the angiogenesis-related genes ANG1, ANG2 and VEGF in human glioma tissues of different WHO pathological grades

In glioma tissue, ANG1, ANG2, and VEGF are expressed in vascular endothelial cells and tumor cells (Figure 1) (Table 3). A significant difference in ANG1 expression was not observed between tumor tissues (32/79) and non-tumor tissues (34/79) (*P* > 0.05). However, significantly higher ANG1 expression was detected in grade I/II (23/38) glioma tissues (*P* < 0.001) than in grade III/IV (9/41) tumors.

**Table 3:** Relationship between expression of ANG 1, ANG 2, VEGF and pathological features of glioma.

**Sample**	***N***	**Positive rate**	***P***
**ANG1**			
Peritumoral tissues	**79**	34 (43.04%)	
Tumor	**79**	32 (40.51%)	*P* = 0.747 > 0.05
**ANG1**			
I/II grades	**38**	23 (60.35%)	
III/IV grades	**41**	9 (21.95%)	*P* < 0.01
**ANG2**			
Peritumoral tissues	**79**	15 (18.99%)	
Tumor	**79**	49 (62.03%)	*P* < 0.01
**ANG2**			
I/II grades	**38**	11 (28.95%)	
III/IV grades	**41**	38 (92.68%)	*P* < 0.01
**VEGF**			
Peritumoral tissues	**79**	22 (27.85%)	
Tumor	**79**	59 (74.68%)	*P* < 0.01
**VEGF**			
I/II grades	**38**	23 (60.53%)	
III/IV grades	**41**	36 (87.80%)	*P* < 0.05

The expression of ANG2 in glioma tissue (48/79) and non-tumor tissue (15/79) was significantly different (*P* < 0.05). Moreover, this difference was also observed in glioma tissues of different pathological grades. A significant difference in ANG2 expression was observed between grade I/II glioma (10/38) and grade III/IV glioma (38/41) (*P* < 0.001).

The expression of VEGF in glioma tissue was similar to Ang2, and a significant difference (*P* < 0.05) was observed between tumor tissues (59/79) and peritumor tissues (22/79). VEGF expression in grade III/IV tissues (36/41) was different from its expression in grade I/II glioma tissue (23/38) (*P* < 0.05).

### The correlation between BMAL1 expression and the expression of ANG1, ANG2, VEGF and HIF-1a

**Figure 2:**
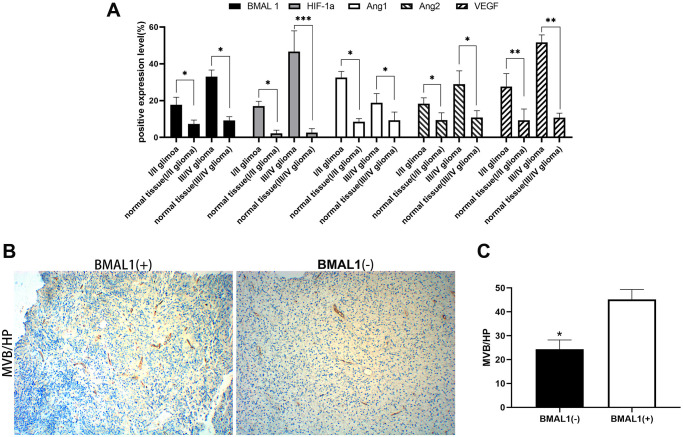
**Expression analysis of BMAL1 and CD34.** (**A**) ANOVA test was used for comparisons the expression of BMAL1 in glioma and normal tissues with different pathological grades. (**B**) A set of representative graphs displaying the number of microvessels in gliomas by labeling CD34. (**C**) Two-sample *t*-test was used for comparisons between the two groups.^*^p < 0.05, ^**^p < 0.01. ^***^p < 0.001.

In this study, 17.72% (14/79) of samples were BMAL1^−^/ANG1^+^, a significant difference was observed compared with BMAL1^+^/ANG1^+^ samples, 53.16% (42/79) (*P* < 0.05), and the expression of the two was negatively correlated (R = −0.365, *P* < 0.001). Notably, 55.7% (44/79) of samples were BMAL1^+^/ANG2^+^, which was significantly higher than the percentage of BMAL1^−^/ANG1^+^ samples (7.59%, 6/79), and the difference was significant (*P* < 0.05); a significant positive correlation was observed between the two (R = 0.402, *P* < 0.001). 63.29% (50/79) of samples were BMAL1^+^/VEGF^+^, a value that is significantly higher than the 11.39% (9/79) of BMAL1^−^/VEGF^+^ samples, and the two were positively correlated (R = 0.397, *P* < 0.001). There was a significant difference between BMAL1^−^/HIF-1a^+^ samples (13.92%,11/79) and BMAL1^+^/HIF-1a^+^ samples (70.88%, 56/79) (*P* < 0.01), and the two were also positively correlated (R = 0.675, *P* < 0.001). These data indicated that increased expression of BMAL1 may promote the expression of HIF-1a, ANG2 and VEGF in glioma (Figure 2A).

### Correlation between expression and the tumor microvascular density

CD34 was used to label the endothelial cells in tumor tissues and determine the microvascular density. The MVD (Microvascular density) was measured in the tumor using the Weidner method [20]. In gliomas, the MVD of BMAL1^−^ samples was 25.3 ± 10.93 and the MVD of and BMAL1^+^ samples was 43.75 ± 10.94, with a statistically significant difference (*P* < 0.05) (Figure 2B and 2C). In gliomas, BMAL1 expression was significantly and positively correlated with the tumor MVD (R = 0.915, *P* < 0.001), suggesting that BMAL1 is closely related to angiogenesis in glioma (Table 4).

**Table 4:** Correlation between BMAL1 expression and MVD.

**BMAL1 Expression**	***n***	**MVD (+)/HP**	**R**	***P***
−	20	25.3 ± 10.93		
+	59	43.75 ± 10.94	0.915	*P* < 0.001

Notes: HP: high power field.

### Correlation of BMAL1 expression with clinical cerebral edema

**Figure 3:**
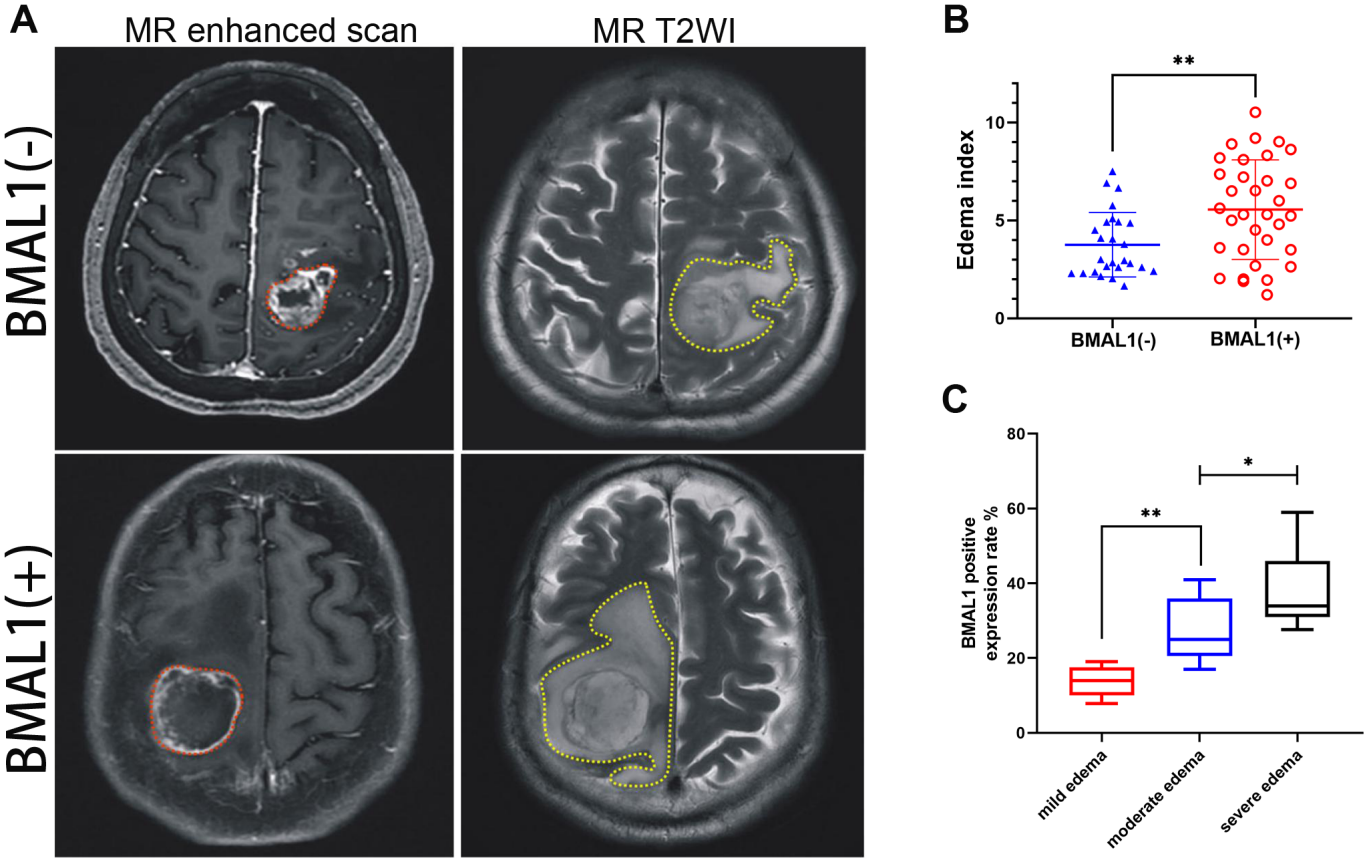
**MR images of clinical patients showed the relationship between peritumoral brain edema and BMAL1 expression.** (**A**) A set of representative graphs displaying MR images of brain edema. The red dotted line is the solid part of the tumor, and the yellow dotted line is the area of brain edema around the tumor. Patients with BMAL1 (+) had obvious peritumoral edema. (**B**) The EI was compared between the BAML1 (+) expression group (*n* = 59) and the BAML1 (−) expression group (*n* = 20). (**C**) BMAL1 expression levels in the mild edema group (*n* = 5), moderate edema group (*n* = 15), and severe edema group (*n* = 39) were assessed. ^*^p < 0.05, ^**^p < 0.01. ^***^p < 0.001.

According to the EI (edema index), we performed peritumor cerebral edema grading in 79 patients with glioma [21]. The median EI was 4.79 (range, 1.20–10.52): 0 with no edema, 16 (20.3%) with mild edema, 26 (32.9%) with moderate edema, and 37 (46.8%) with severe edema (Table 5). Although clinical MRI scans suggested that all patients had peritumor brain edema to varying degrees, compared with the BMAL1^−^ patients, patients with BMAL1^+^ expression were more likely to exhibit moderately severe or severe brain edema (Figure 3A and 3B). Furthermore, we analyzed the BMAL1 levels in different edema groups. The BMAL1 expression level in the moderate edema group was higher than that in the mild edema group (*P* < 0.01), and the expression level in the severe edema group was higher than that in moderate edema group (*P* < 0.05) (Figure 3C).

**Table 5:** Correlation between BMAL1 expression and edema.

**BMAL1 Expression**	***n***	**Edema**		**X^2^**	***P***
		+	++/+++		
+	59	5	54		
−	20	11	9	20.02	<0.0001

Notes: +: mild edema (1 < EI≤ 1.5); ++: moderate edema (1.5 < EI≤ 3); +++: severe edema (EI > 3).

### The regulatory effect of BMAL1 expression on HIF-1a, ANG1, ANG2 and VEGF expression

**Figure 4:**
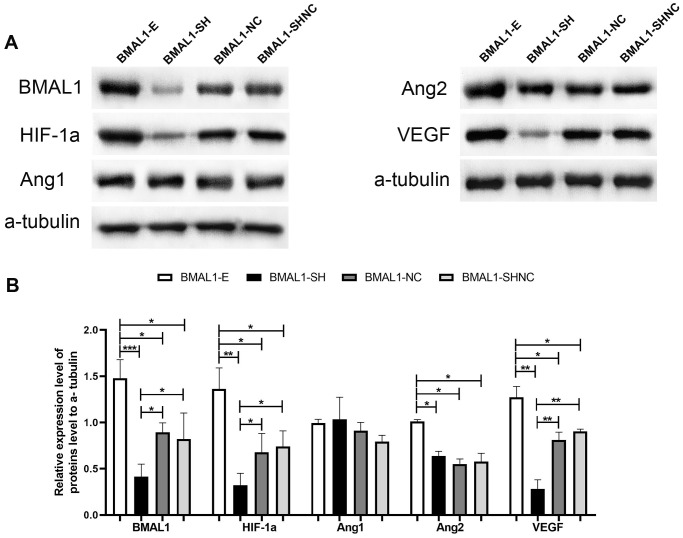
**The regulatory pathway of BMAL1 on proangiogenic factors was assessed by western blot.** (**A**) A set of representative graphs displaying the regulatory pathway of BMAL1 in glioma cells. (**B**) Two-sample *t*-test was used for comparisons between the two groups. ^*^p < 0.05, ^**^p < 0.01. ^***^p < 0.001.

After the silencing of the *BMAL1*, the expression of VEGF and HIF-1a were significantly decreased in the BMAL1-Sh group compared with the BMAL1-NC group and the BMAL1-Sh NC group (Figure 4A and 4B); however, a similar result did not found in the expression of ANG2, compared with the BMAL1-NC group (*P* = 0.156 > 0.05) and the BMAL1-Sh NC group (*P* = 0.239 > 0.05); a significant difference in ANG1 expression was not observed compared with the BMAL1-NC group (*P* = 0.193 > 0.05) and BMAL1-Sh NC group (*P* = 0.218 > 0.05). A subtle difference expression pattern was observed in glioma cells that overexpressed BMAL1. In other words, after the overexpression of the BMAL1 gene, the expression of HIF-1a, ANG2 and VEGF was correspondingly increased compared with the BMAL1-NC group and the BMAL1-SHNC group (Figure 4A and 4B). However, no difference in expression of ANG1 was observed compared with the BMAL1-NC group (*P* = 0.203 > 0.05) and the BMAL1-SHNC group (*P* = 0.114 > 0.05).

## DISCUSSION

Glioma is considered an angiogenesis-dependent tumor [22]. A serious imbalance between vascular growth-promoting and inhibitory factors in the glioma microenvironment has been observed [23]. As previously reported, *ANG1*, *ANG2* and *VEGF* are expressed at high levels in gliomas, and significant differences in their expression were observed in different pathological grades. Therefore, anti-angiogenic therapy for gliomas should be promising; however, in fact, glioma is one of the tumors in which anti-angiogenesis therapy displays poor efficacy [9]. Based on these findings, a single target therapy targeting a specific vascular factor may not improve the angiogenesis and vascular structure of glioma, or other more complex regulatory mechanisms may regulate angiogenesis in glioma.

High *BMAL1* expression in tumor cells was associated with poor clinical outcomes in patients with colorectal cancer receiving Beva (anti-VEGF) combined with chemotherapy [10]. Furthermore, this resistance to Beva was caused by an internal, intracellular "escape" pathway of the tumor cells that involved *BMAL1*. This pathway initiates the continuous synthesis and secretion of VEGF, followed by continued stimulation of the VEGFA/VEGFR2 signaling pathway through autocrine/paracrine mechanisms [10]. Lasse et al. also confirmed that *BMAL1* regulated angiogenesis through the *VEGF* pathway in zebrafish [17]. Thus, rhythm genes may contribute to glioma tolerance to vascular targeted therapy. In addition to *VEGF*, *BMAL1* was also positively correlated with the expression of *ANG2* in glioma in our study. It has been demonstrated that the hypoxic microenvironment of glioma could up-regulate the expression of *ANG2* and *VEGF* via *HIF-1a* pathway [19, 24]. The above-mentioned findings prompted us to investigate the correlation between HIF-1a and BMAL1 expression in glioma. Our IHC results showed that the intensity of HIF-1a positive expression was positively correlated with BMAL1 expression. Additionally, the expression of BMAL1 correlated with the pathological grade of glioma, but not with the patient's age, gender, or tumor size. Therefore, *BMAL1* expression may be independent of the initial origin of glioma, but is related to the later development of glioma, and *BMAL1* is likely to participate in the mechanism regulating glioma angiogenesis and we hypothesized that *BMAL1* may up-regulate the expression of *VEGF* and *ANG2* through the *HIF-1a* pathway.

To more directly assess the impact of *BMAL1* on the expression levels of *HIF-1a*, *ANG2* and *VEGF* we used primary glioma cells to construct cell models with *BMAL1* overexpression and silencing and to determine whether *BMAL1* is involved in *HIF-1a*, *ANG2* and *VEGF* expression in gliomas. In our study, when the *BMAL1* was up-regulated, *HIF-1a*, *ANG2* and *VEGF* expression also increased; when *BMAL1* expression was silenced, *HIF-1a* and *VEGF* expression decreased, while the expression of *ANG1*, *ANG2* was not affected. It is a fascinating result that *HIF-1a*, *VEGF* increases or decreases with the increase or silencing of *BMAL1* expression. Thus, *BMAL1* may be involved in regulating the expression of *VEGF*, *HIF-1a*. This result is consistent with the findings reported by Lasse and colleagues who found that the *VEGF* expression pattern matched the expression pattern of *BMAL1* and *BMAL1* regulated the transcription of *VEGF* through E-boxes [25]. However, it is worth noting that *ANG2* increases with the increase of *BMAL1* expression but does not decrease with the silencing of *BMAL1* expression. It has been previously reported that hypoxia can up-regulate the expression of *ANG2*, and the hypoxia regulation of *ANG2* is *HIF-1a* dependent [19]. Therefore, upregulation of *ANG2* expression associated with *BMAL1* overexpression may be related to elevated *HIF-1a* expression. The expression of *ANG2* was not affected by the silent expression of *BMAL1*, indicative of the regulation of *ANG2* expression may be multifactorial. From our experiment, we can speculate that *BMAL1* may be one of the main factors regulating *ANG2* expression. Still, it is not the only factor, and its specific molecular pathway needs to be clarified by more experiments.

The abnormal expression of *ANG2* and *VEGF* is the main explanation for the presence of a large number of heterogeneous blood vessels in tumor tissues [26]. Dual antagonists targeting ANG2/VEGF effectively improve the vascular structure and function and reduce peritumor brain edema in patients with glioma [25, 27]. In the present study, *BMAL1* expression was positively correlated with the expression of *ANG2* and *VEGF*, and thus we speculated that high *BMAL1* expression might be correlated with the microvascular density of glioma and the degree of peritumor brain edema. We verified our hypothesis by analyzing human glioma tissue to determine the correlation between *BMAL1* expression and microvessels, and performed MRI (Magnetic Resonance Imaging) to understand the correlation between the expression of the *BMAL1* gene and peritumor edema. The positive expression of *BMAL1* protein in tumor tissues correlated with the distribution of high MVD, which was more obvious in high-grade glioma. Similar results were also obtained using MRI to assess brain edema; the higher the positive rate of *BMAL1* expression, the more severe the brain edema. Based on these results, *BMAL1* may plays an important role in brain tumor microangiogenesis and peritumor edema.

In summary, we speculated that *BMAL1* might be involved in angiogenesis in human glioma cells by regulating *VEGF* and *ANG2* expression via *HIF-1a* pathway. Researchers have not determined whether *BMAL1* regulates tumor biological behaviors by regulating angiogenesis in glioma. However, our results support the hypothesis that *BMAL1* is involved in glioma angiogenesis by regulating the *ANG2*/*VEGF* pathway and affects the degree of peritumor brain edema. Although the specific molecular pathway is not yet clear, this discovery will be beneficial to developing new clinical drugs for glioma and obtaining a better understanding of the mechanism underlying the development of peritumor edema, providing new insights into the treatment of peritumor brain edema.

## MATERIALS AND METHODS

### Clinical samples

All the specimens were collected at the Neurosurgery Department of Jingmen First People's Hospital affiliated with Hubei Minzu University from January 2017 to October 2020. None of the patients had received preoperative radiotherapy or chemotherapy. The specimens were removed from 12:00 noon to before 3:00 p.m., and 79 glioma specimens were collected. The patients were 17–69 years old. The patients included 42 males and 37 females. According to WHO glioma grading criteria, 38 cases of low-grade astrocytoma (grade I/II) and 41 cases (grade III/IV) of anaplastic astrocytoma and glioblastoma multiforme were obtained. Nonneoplastic tissue samples were collected from 79 patients. Some of the specimens were prepared as paraffin sections for immunohistochemistry, and primary glioma cells were extracted from other specimens within one hour.

### Primary glioma cell isolation and culture

Within an hour of obtaining fresh specimens of glioma tissues (WHO class III/IV), the samples were transferred on ice to the ultra-clean cabinet, minced, rinsed with saline repeatedly, centrifuged, and the supernatant was digested with II collagenase at 37°C until most of the tissue block was dissociated. DMEM containing 15% fetal bovine serum (FBS) was added to terminate the digestion, cells were filtered through a 70 μm cell mesh filter, centrifuged, the supernatant was discarded, and pellets were resuspended and cultured in DMEM.

### MRI examination and edema index measurement

Patients underwent an MRI plain scan and contrast-enhanced scan before the operation with a Siemens 3.0 T MR machine. The protocol of Qu et al. [19] was used to examine MRI and measure the edema index. All MR imaging data were measured by a computer workstation. Preoperative T1-enhanced TSE sequence of MRI was employed to measure tumor volume. Specifically, the first step was to measure the axial maximum diameter (A), coronal maximum diameter (B), and sagittal maximum diameter (C) of the tumor on MR imaging T1-enhanced TSE sequence (TR = 2000 ms; TE = 9 ms; section thickness = 6 mm; FOV = 230 × 187 mm; voxel size = 0.7 × 0.7 × 6.0 mm) in each patient. The second step is to calculate the tumor volume according to the formula previously reported: V = 4/3π × A/2 × B/2 × C/2 [28]. The volume of perituminal brain edema was measured using T2-weighted and FLAIR TSE sequences, with specific MRI operating parameters as follows: TR = 9000 ms; TE = 81 ms; section thickness = 6 mm; FOV = 230 × 200 mm; voxel size = 0.7 × 0.7 × 6.0 mm. As well as measuring tumor volume, we measured the maximum diameter of cerebral edema in axial, coronal, and sagittal scans and calculated the edema volume. The edema index (EI) was used to evaluate the degree of peritumoral edema, and its calculation formula was as follows: EI = (V tumor + edema)/(V tumor). According to previous reports, peritumoral edema can be divided into four degrees according to the edema index [29], as follow: no edema (EI = 1), mild edema (1 < EI≤ 1.5), moderate edema (1.5 < EI≤ 3), and severe edema (EI > 3).

### Immunohistochemistry

Immunohistochemistry (IHC) was performed using the SP method. The specimens were prepared as paraffin sections, baked, dewaxed and hydrated, subjected to dehydration with a gradient of ethanol solution, and subjected to the blocking of endogenous peroxidase activity, antigen repair (high-pressure repair), washing, sealing, and an incubation with BMAL1 (ab231793, ABCAM), HIF-1a (ab114977, ABCAM), ANG1 (ab133425, ABCAM), ANG2 (Ab155106, ABCAM), VEGF (ab32152, ABCAM) and CD34 (ab8158, ABCAM) primary antibodies at 4°C overnight. Next, sections were incubated with secondary antibodies, DAB was used for color development, and sections were stained with hematoxylin, dehydrated, sealed, and observed under a microscope.

### Construction of BMAL1 overexpression and silencing vectors

Synthetic primers were designed based on the BMAL 1 gene sequence in NCBI. The following primer sequences were used: upstream lead 5′-CCGGAAT TCGCCACCATGGCAGACCAGAGAATGGACATTT CTTC-3; downstream primers: 5′-CCGGGATCCT TACAGCGGCCATGGCAAGTC-3′. Under the action of T4 DNA ligase, the lentivirus vector PGMLV-PA6 was connected to the BMAL1 fragment.

Based on the sequence of the BMAL1 gene, a synthetic shRNA oligomeric single-stranded DNA with the following sequence (5′ to 3′) designed: BMAL1 sh3T: GATCCGCACGCGATAGATGGAAAGTTCTCGAGA ACTTTCCATCTATCGCGTGCTTTTTT and BMAL1 sh3B: AATTAAAAAAGCACGCGATAGATGGAA AGTTCTCGAGAACTTTCCATCTATCGCGTGCG. The oligomers were annealed to form a double-stranded structure. Under the action of T4 DNA ligase, the lentivirus vector PGMLV-SB3 was connected with the double-stranded primer. The following steps were conducted according to the manufacturer’s instructions and products were stored at –80°C.

### Transfection and western blotting

The virus stock was diluted to an appropriate MOI (multiplicity of infection), and the diluted lentivirus was added to the primary cultured cells. Puromycin was used to screen stably transfected cells. Finally, the following four groups were obtained: the BMAL1 overexpression group (BMAL1-E), BMAL1 overexpression control group (BMAL1-NC), BMAL1 silent expression group (BMAL1-SH), and BMAL1 silent expression control group (BMAL1-SHNC). Cells from these experimental groups were cultured in 6 well plate. After reaching 70% confluence, the cells were lysed on ice using RIPA buffer. After protein extraction, the protein samples were loaded in the wells of SDS-PAGE gels and separated. The expression of target proteins BMAL1 (ab231793, ABCAM), HIF-1a (ab114977, ABCAM), ANG1 (ab133425, ABCAM), ANG2 (Ab155106, ABCAM) and VEGF (ab32152, ABCAM) was detected by Western blot.

### Ethics statement for human specimens

All studies on human subjects were approved by Hubei University for Nationalities Ethics Committee. The ethical approval number for excised material is HBMZ-2012018.

### Statistical analysis

The association between tumor grade (high-grade/low-grade gliomas) and expression of the investigated proteins (negative/positive) was assessed using the Chi-Square Test and Two-sample *t*-test, included in the Statistical Package for the Social Science, version 13.0. Using Kendall’s tau-b Correlation to analyse correlation between the expression of BMAL1 and ANG1, ANG2, VEGF and edema index. Using Spearman Correlation to analyse correlation between the expression of BMAL1 and MVD.

### Data availability

The datasets generated during and/or analysed during the current study are available from the corresponding author on reasonable request.

## References

[1] Louis DN, Perry A, Reifenberger G, von Deimling A, Figarella-Branger D, Cavenee WK, Ohgaki H, Wiestler OD, Kleihues P, Ellison DW. The 2016 World Health Organization Classification of Tumors of the Central Nervous System: a summary. Acta Neuropathol. 2016; 131:803–20. 10.1007/s00401-016-1545-1 27157931

[2] Bottsford-Miller JN, Coleman RL, Sood AK. Resistance and escape from antiangiogenesis therapy: clinical implications and future strategies. J Clin Oncol. 2012; 30:4026–34. 10.1200/JCO.2012.41.9242 23008289 PMC3488272

[3] Jayson GC, Hicklin DJ, Ellis LM. Antiangiogenic therapy--evolving view based on clinical trial results. Nat Rev Clin Oncol. 2012; 9:297–303. 10.1038/nrclinonc.2012.8 22330688

[4] Sorensen AG, Batchelor TT, Zhang WT, Chen PJ, Yeo P, Wang M, Jennings D, Wen PY, Lahdenranta J, Ancukiewicz M, di Tomaso E, Duda DG, Jain RK. A "vascular normalization index" as potential mechanistic biomarker to predict survival after a single dose of cediranib in recurrent glioblastoma patients. Cancer Res. 2009; 69:5296–300. 10.1158/0008-5472.CAN-09-0814 19549889 PMC2824172

[5] Jain RK. Normalizing tumor vasculature with anti-angiogenic therapy: a new paradigm for combination therapy. Nat Med. 2001; 7:987–89. 10.1038/nm0901-987 11533692

[6] Griscelli F, Li H, Cheong C, Opolon P, Bennaceur-Griscelli A, Vassal G, Soria J, Soria C, Lu H, Perricaudet M, Yeh P. Combined effects of radiotherapy and angiostatin gene therapy in glioma tumor model. Proc Natl Acad Sci U S A. 2000; 97:6698–703. 10.1073/pnas.110134297 10823901 PMC18707

[7] Miletic H, Niclou SP, Johansson M, Bjerkvig R. Anti-VEGF therapies for malignant glioma: treatment effects and escape mechanisms. Expert Opin Ther Targets. 2009; 13:455–68. 10.1517/14728220902806444 19335067

[8] Osswald M, Jung E, Sahm F, Solecki G, Venkataramani V, Blaes J, Weil S, Horstmann H, Wiestler B, Syed M, Huang L, Ratliff M, Karimian Jazi K, et al. Brain tumour cells interconnect to a functional and resistant network. Nature. 2015; 528:93–98. 10.1038/nature16071 26536111

[9] Wick W, Platten M, Wick A, Hertenstein A, Radbruch A, Bendszus M, Winkler F. Current status and future directions of anti-angiogenic therapy for gliomas. Neuro Oncol. 2016; 18:315–28. 10.1093/neuonc/nov180 26459812 PMC4767238

[10] Burgermeister E, Battaglin F, Eladly F, Wu W, Herweck F, Schulte N, Betge J, Härtel N, Kather JN, Weis CA, Gaiser T, Marx A, Weiss C, et al. Aryl hydrocarbon receptor nuclear translocator-like (ARNTL/BMAL1) is associated with bevacizumab resistance in colorectal cancer via regulation of vascular endothelial growth factor A. EBioMedicine. 2019; 45:139–54. 10.1016/j.ebiom.2019.07.004 31300350 PMC6642438

[11] Reszka E, Przybek M, Muurlink O, Pepłonska B. Circadian gene variants and breast cancer. Cancer Lett. 2017; 390:137–45. 10.1016/j.canlet.2017.01.012 28109907

[12] Okazaki F, Matsunaga N, Okazaki H, Azuma H, Hamamura K, Tsuruta A, Tsurudome Y, Ogino T, Hara Y, Suzuki T, Hyodo K, Ishihara H, Kikuchi H, et al. Circadian Clock in a Mouse Colon Tumor Regulates Intracellular Iron Levels to Promote Tumor Progression. J Biol Chem. 2016; 291:7017–28. 10.1074/jbc.M115.713412 26797126 PMC4807285

[13] Khan S, Liu Y, Siddique R, Nabi G, Xue M, Hou H. Impact of chronically alternating light-dark cycles on circadian clock mediated expression of cancer (glioma)-related genes in the brain. Int J Biol Sci. 2019; 15:1816–34. 10.7150/ijbs.35520 31523185 PMC6743288

[14] Sulli G, Lam MTY, Panda S. Interplay between Circadian Clock and Cancer: New Frontiers for Cancer Treatment. Trends Cancer. 2019; 5:475–94. 10.1016/j.trecan.2019.07.002 31421905 PMC7120250

[15] Masri S, Sassone-Corsi P. The emerging link between cancer, metabolism, and circadian rhythms. Nat Med. 2018; 24:1795–803. 10.1038/s41591-018-0271-8 30523327 PMC6535395

[16] Puram RV, Kowalczyk MS, de Boer CG, Schneider RK, Miller PG, McConkey M, Tothova Z, Tejero H, Heckl D, Järås M, Chen MC, Li H, Tamayo A, et al. Core Circadian Clock Genes Regulate Leukemia Stem Cells in AML. Cell. 2016; 165:303–16. 10.1016/j.cell.2016.03.015 27058663 PMC4826477

[17] Jensen LD, Cao Y. Clock controls angiogenesis. Cell Cycle. 2013; 12:405–408. 10.4161/cc.23596 23324349 PMC3587440

[18] Li A, Lin X, Tan X, Yin B, Han W, Zhao J, Yuan J, Qiang B, Peng X. Circadian gene Clock contributes to cell proliferation and migration of glioma and is directly regulated by tumor-suppressive miR-124. FEBS Lett. 2013; 587:2455–60. 10.1016/j.febslet.2013.06.018 23792158

[19] Simon MP, Tournaire R, Pouyssegur J. The angiopoietin-2 gene of endothelial cells is up-regulated in hypoxia by a HIF binding site located in its first intron and by the central factors GATA-2 and Ets-1. J Cell Physiol. 2008; 217:809–18. 10.1002/jcp.21558 18720385

[20] Weidner N. Current pathologic methods for measuring intratumoral microvessel density within breast carcinoma and other solid tumors. Breast Cancer Res Treat. 1995; 36:169–80. 10.1007/BF00666038 8534865

[21] Qu S, Hu T, Qiu O, Su Y, Gu J, Xia Z. Effect of Piezo1 Overexpression on Peritumoral Brain Edema in Glioblastomas. AJNR Am J Neuroradiol. 2020; 41:1423–29. 10.3174/ajnr.A6638 32675337 PMC7658862

[22] Onishi M, Ichikawa T, Kurozumi K, Date I. Angiogenesis and invasion in glioma. Brain Tumor Pathol. 2011; 28:13–24. 10.1007/s10014-010-0007-z 21221826

[23] Osada H, Tokunaga T, Hatanaka H, Kawakami T, Tsuchida T, Abe Y, Tsugu A, Kijima H, Yamazaki H, Shima K, Osamura Y, Ueyama Y, Nakamura M. Gene expression of angiogenesis related factors in glioma. Int J Oncol. 2001; 18:305–309. 10.3892/ijo.18.2.305 11172596

[24] Nicolas S, Abdellatef S, Haddad MA, Fakhoury I, El-Sibai M. Hypoxia and EGF Stimulation Regulate VEGF Expression in Human Glioblastoma Multiforme (GBM) Cells by Differential Regulation of the PI3K/Rho-GTPase and MAPK Pathways. Cells. 2019; 8:1397. 10.3390/cells8111397 31698752 PMC6912653

[25] Solecki G, Osswald M, Weber D, Glock M, Ratliff M, Müller HJ, Krieter O, Kienast Y, Wick W, Winkler F. Differential Effects of Ang-2/VEGF-A Inhibiting Antibodies in Combination with Radio- or Chemotherapy in Glioma. Cancers (Basel). 2019; 11:314. 10.3390/cancers11030314 30845704 PMC6468722

[26] Fidler IJ, Ellis LM. The implications of angiogenesis for the biology and therapy of cancer metastasis. Cell. 1994; 79:185–88. 10.1016/0092-8674(94)90187-2 7525076

[27] Kloepper J, Riedemann L, Amoozgar Z, Seano G, Susek K, Yu V, Dalvie N, Amelung RL, Datta M, Song JW, Askoxylakis V, Taylor JW, Lu-Emerson C, et al. Ang-2/VEGF bispecific antibody reprograms macrophages and resident microglia to anti-tumor phenotype and prolongs glioblastoma survival. Proc Natl Acad Sci U S A. 2016; 113:4476–81. 10.1073/pnas.1525360113 27044098 PMC4843473

[28] Osawa T, Tosaka M, Nagaishi M, Yoshimoto Y. Factors affecting peritumoral brain edema in meningioma: special histological subtypes with prominently extensive edema. J Neurooncol. 2013; 111:49–57. 10.1007/s11060-012-0989-y 23104516

[29] Chinese Neurosurgical Society. [Consensus on the drug treatment of peritumoral brain edema]. Zhonghua Yi Xue Za Zhi. 2010; 90:5–9. 20356516

